# Typical Clinical Presentation of an Autosomal Dominant Polycystic Kidney Disease Patient with an Atypical Genetic Pattern

**DOI:** 10.3390/genes16010039

**Published:** 2024-12-30

**Authors:** Nenzi Marzano, Carlotta Caprara, Thiago Reis, Diego Pomarè Montin, Sofia Maria Pretto, Matteo Rigato, Anna Giuliani, Fiorella Gastaldon, Barbara Mancini, Claudio Ronco, Monica Zanella, Daniela Zuccarello, Valentina Corradi

**Affiliations:** 1 The International Renal Research Institute of Vicenza (IRRIV) Foundation, ULSS 8 BERICA, San Bortolo Hospital, 36100 Vicenza, Italy; nenzimarzano@yahoo.it (N.M.); carlotta.caprara@aulss8.veneto.it (C.C.); thiagoreisnefro@gmail.com (T.R.); diegopomarem@hotmail.it (D.P.M.); sofiamariapretto@gmail.com (S.M.P.); matteo.rigato@aulss8.veneto.it (M.R.); cronco@goldnet.it (C.R.); monica.zanella@aulss8.veneto.it (M.Z.); valentina.corradi@aulss8.veneto.it (V.C.); 2 Department of Nephrology, Dialysis and Transplantation, ULSS 8 BERICA, San Bortolo Hospital, 36100 Vicenza, Italy; anna.giuliani@aulss8.veneto.it (A.G.); fiorella.gastaldon@aulss8.veneto.it (F.G.); 3 Department of Medical Genetics and Genomics, ULSS 8 BERICA, San Bortolo Hospital, 36100 Vicenza, Italy; barbara.mancini@aulss8.veneto.it; 4 Laboratory of Molecular Pharmacology, Faculty of Health Sciences, University of Brasília, Brasília 70910-900, Brazil; 5 Fenix Nephrology, São Paulo 04508-011, Brazil; 6 Hospital Sírio-Libanês, São Paulo 01308-050, Brazil; 7 Department of Health Sciences, University of Florence, 50121 Florence, Italy

**Keywords:** polycystic kidney disease, chronic kidney disease, mosaicism, next-generation sequencing, multiple ligation-dependent probe amplification

## Abstract

**Background**: Autosomal Dominant Polycystic Kidney Disease (ADPKD) is mainly characterized by renal involvement with progressive bilateral development of renal cysts and volumetric increase in the kidneys, causing a loss of renal function, chronic kidney disease (CKD), and kidney failure. The occurrence of mosaicism may modulate the clinical course of the disease. Mosaicism is characterized by a few cell populations with different genomes. In these special cases, a genetic diagnosis could be challenging. **Methods**: Herein, we describe the case of a 47-year-old woman presenting with typical ultrasound and computed tomography features of ADPKD. She had stage 3b CKD and hypertension. There was no family history of ADPKD, prompting an investigation with a genetic test. Target next-generation sequencing (NGS) did not detect the presence of any genomic variants. Therefore, we carried out second-level genetic analysis to investigate the presence of a large rearrangement through a multiple ligation-dependent probe amplification (MLPA) analysis of PKD1 and PKD2 genes. **Results**: MLPA showed a large deletion (portion including exons 2–34 of PKD1) present in the heterozygosis with a percentage of cells close to the resolution limits of the technique used (<25–30%). We concluded that the large deletion identified was mosaicism. This variant is not reported in major ADPKD databases, but due to the type of mutation and the patient’s clinical picture, it should be considered as likely pathogenic. **Conclusions**: A stepwise genetic approach might be useful in those cases where standard methods do not allow one to reach a definitive diagnosis.

## 1. Introduction

Polycystic kidney diseases (PKD) are a heterogeneous group of diseases. Among these, we highlight a group of pathologies that are also heterogeneously characterized by anomalies affecting a structure called the primary cilium and are collectively known as ciliopathies [1]. Based on the type of inheritance, we can distinguish between autosomal dominant polycystic kidney disease (ADPKD) and autosomal recessive polycystic kidney disease (ARPKD) [2]. ADPKD is considered to be the most common genetic kidney disorder and is the fourth leading cause of chronic kidney disease (CKD) requiring renal replacement therapy [2]. It affects 12.5 million individuals worldwide, regardless of ethnicity or gender, with an incidence of 1 in every 400 to 1000 live births [3]. Population-based studies have estimated the prevalence of ADPKD to be much lower, noting that it does not exceed the limit of 5: 10,000 inhabitants and that it would be compatible with the definition of rare disease adopted by the European Medicines Agency and the Food and Drug Administration [4]. ADPKD is mainly characterized by renal involvement with the bilateral, age-dependent progressive development of renal cysts and an increase in the volume of the kidneys leading to the progressive loss of renal function, the development of CKD, and kidney failure [5]. In general, ADPKD has a late onset, typically in adulthood, between the third and fourth decades of life [6,7]. Nonetheless, some patients have an early onset and rapid disease progression. Despite the variability of onset, ADPKD has complete penetrance.

The treatments that are currently applied aim to reduce the progression of CKD. Tolvaptan is the only medication approved by the Italian Drug Agency (AIFA) and by the European Medicines Agency (EMA). This drug has shown significant efficacy in preserving kidney function and slowing down the progression of kidney failure [8]. Notably, patients without a family history of ADPKD must undergo genetic testing to confirm the disease and become candidates for this treatment [9]. ADPKD is transmitted with an autosomal dominant trait, without generational leaps and distinction of gender. In 10% of cases, it occurs de novo [10]. Roughly three-quarters of the resolved cases of ADPKD are attributable to mutations in PKD1 (16p13.3, OMIM #173900) [11] while 15–18% are attributable to PKD2 (4q21-23, OMIM #61395) [11]. Other genes have recently been found as potentially implicated with ADPKD, such as GANAB (<0.5%, 11q12.3, OMIM #600666) [12], DNAJB11 (<0.5%, 3q27.3, OMIM #618061) [13,14], IFT140 (1–2%, 16p13.3, OMIM #614620) [15], ALG5 (<0.5%, 13q13.3, OMIM #620056) [16], and ALG 9 (<0.5%, 11q23.1, OMIM #606941) [17]. In addition to these atypical genes, ~ 5% of cases remain genetically unresolved (GUR) [18]. The progression of the disease is highly variable, due in part to a strong gene locus effect [19,20] and an allelic effect. The Genkyst study confirms the gene effect and shows the effect of the mutation type on the phenotype [21]. Patients with truncating PKD1 mutations progress to kidney failure at the median age of 52 years (95% CI: 51.2–53.9) compared to the median age of 80 years (95% CI: 77.1 to 82.8) in patients with mutations in the PKD2 gene [22].

ADPKD diagnosis relies mainly on the use of imaging techniques such as ultrasound, computed tomography (CT), or magnetic resonance imaging (MRI) [7,23]. In selected cases, it is necessary to carry out genetic testing to confirm the diagnosis [24,25]. The development of NGS methodologies and the resulting expansion of clinical genetic testing have decreased costs and improved availability. The next-generation approach is increasingly used, avoiding more laborious techniques such as Sanger sequencing [24,26,27] and multiplex ligation-dependent probe amplification (MLPA) [24]. Diagnosis usually requires the successive application of different techniques to provide a diagnosis that is as safe and accurate as possible. Although Whole-Genome Sequencing (WGS) and Whole-Exome Sequencing (WES) are increasingly used as first-line diagnostic tools for various diseases, in the diagnostic analysis of ADPKD, the characteristics of the PKD1 gene (16p13.3) make variant detection particularly challenging. In the genomic region between exon 1 and exon 33, the high sequence homology with known pseudogenes makes the WES approach much less sensitive and specific for identifying pathogenic variants. The approach using targeted sequencing with a gene panel (Targeted NGS testing) is preferred for patients with relatively specific clinical presentations, such as ADPKD [27,28]. However, broader genetic tests, such as WES, continue to be important strategies for atypical or clinically undefined cases [24]. Meanwhile, ADPKD is characterized by a complex inheritance such as hypomorphic mutations in trans-heterozygosity, phenocopies, mosaicism, and by significant phenotypic variability [5].

Mosaicism is a genetic condition in which cells have two or more different genomes within an individual derived from a single zygote. We consider it to be somatic mosaicism when the mutation occurs in somatic cells during embryogenesis. On the other hand, mutation can occur within gonads, resulting in germline mosaicism that can be inherited [29]. Mutations involving both somatic and germline cells are also possible [30]. In some cases, mosaicism may modify the presentation of the disease, and its identification could better define an ADPKD population [31]. Although molecular diagnostic techniques, such as NGS or Fluorescence In Situ Hybridization (FISH), can assist in detecting mosaicism, its identification is burdensome given the absence of a specific technique to detect it.

The presentation of ADPKD can vary significantly within a family, and cases that appear to be de novo may be due to mosaicism. There are few cases of mosaicism in ADPKD described in the literature [31,32,33,34,35]. In individuals with mosaicism, the severity of the disease can range from typical ADPKD to much milder forms. A recent study investigating mosaicism in 20 ADPKD families found that, in 5 of these families, the pathogenic variant was passed on to the next generation, while in the other 15, it was sporadic [31]. When examining kidney size and function, individuals with mosaicism were found to have a milder disease progression compared to a control group of PKD1-ADPKD patients, though only a small number exhibited pronounced asymmetry. It is estimated that mosaicism may occur in at least 1% of ADPKD cases [18,31]. The application of several techniques aimed at the screening and sequencing of multiple cell types belonging to several body parts derived from the three different embryonic leaves may serve as a promising procedure for diagnosing PKD mosaicism [36].

Here, we present an intriguing clinical case in which the choice and strategy of genetic analysis were crucial in determining both the genetic and clinical diagnosis of ADPKD.

## 2. Case Presentation

**Figure 1:**
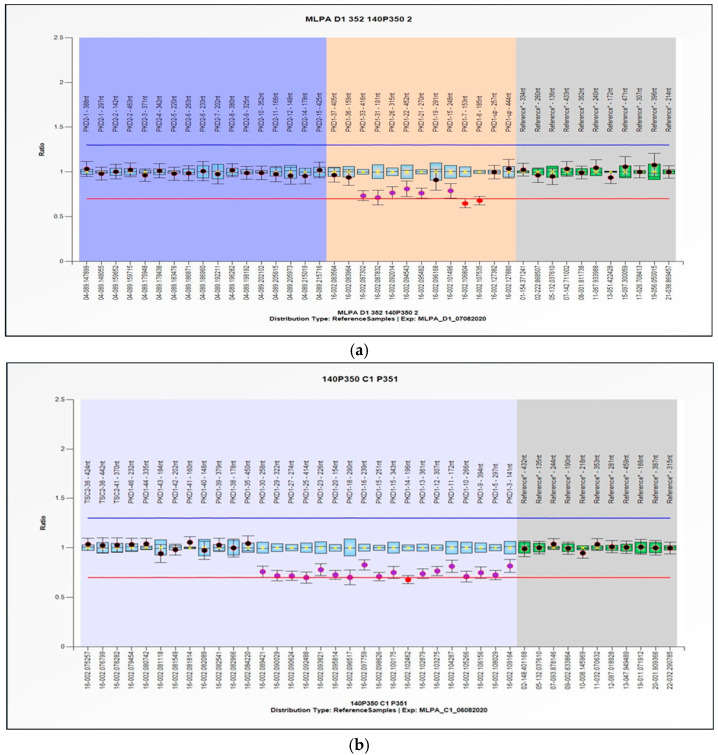
Analysis with multiplex ligation probe amplification (MLPA) of PKD1 gene (light blue and orange background) (NM_001009944.3, except exons 1, 2, 4, 8, 17, 24, 28, 32, 34, 45) and of PKD2 gene (blue background) (NM_000297.4, ecept exon 13). The molecular investigation was performed on genomic DNA using Kit Salsa MLPA ProbeMix P352-D1 PKD1-PKD2 and ProbeMix P351-C1 PKD1 ©MRC Holland. The result shows a deletion of the 3–33 exons of the PKD1 gene in mosaic. The arrangement of probes based on chromosomal position reveals a heterozygous deletion (red line): probe ratio <0.5 (red dots), probe ratio 1 < x < 0.5 (purple dots). The first deleted probe was number 141 on the PKD1 gene (hg18 loc.16-002.109164) (**b**), while the last was number 416 on the PKD1 gene (hg18 loc.16-002.087302) (**a**). Black dots represent boundedprobes; exons are represented by light blue boxes (green boxes are reference exons). Results are displayed by the Coffalyser.Net software (https://www.mrcholland.com/technology/software/coffalyser-net, accessed on 22 December 2024).

A 47-year-old female with a previous diagnosis of CKD stage 3b secondary to ADPKD and hypertension underwent routine follow-up at our outpatient clinic. She had no family history of ADPKD. A genetic confirmation of the condition was required by the regional guideline before she started taking Tolvaptan [9]. The first-level genetic analysis was performed using targeted next-generation sequencing (NGS) which provides a parallel sequencing of millions of DNA fragments, allowing for the analysis of multiple genes simultaneously and shortening the time required for the genetic analysis [37]. The SOPHIA DDM™ Nephropathies Solution (SOPHiA GENETICS, Lausanne, Switzerland) used on the Illumina platform provided amplification and sequencing. The genetic panel covered the coding regions and splicing junctions (±5 bp) of the 44 most relevant genes (target region of 105.8 kb) related to a broad range of nephropathies, including PKD1 and PKD2. In our case, the identification of genomic variants in the ADPKD target genes was negative, despite the typical phenotype of the condition. Notably, in 7% of cases investigated, no variants are detected [38], prompting the use of a second-level genetic analysis to explore the large rearrangement of previously investigated genes PKD1 and PKD2 and, if negative, other genes that have recently been found to be potentially implicated in the disease and that are not present in the NGS gene panel used [12,13,15,16,17]. A molecular investigation was then performed to search for large rearrangements using a multiplex ligation-dependent probe amplification (MLPA) analysis of the PKD1 and PKD2 genes. MPLA is a multiple PCR analysis method for detecting abnormalities in the number of copies in specific and predetermined areas of the genome that may be associated with specific genetic disorders. This analysis revealed a large heterozygous deletion involving the 3–33 exons of PKD1 (Figure 1), observed in mosaic form with a percentage close to the resolution limits of the technique (i.e., 25–30%). The exact boundaries of the deletion could not be established, as some probes were absent from the kit used (SALSA^®^ MLPA^®^ Probemixes P352-D1 PKD1-PKD2 and P351-C1 PKD1, ©MRC Holland, Amsterdam, The Netherlands). The probe ratio for the deleted probes ranged between 1 and 0.5. The value of 0.5 represents the threshold indicating the deletion, and the ratio falling between 1 and 0.5 led us to infer the presence of mosaicism. The variant is not listed in the ADPKD pathology reference database (ADPKD Variant Database, https://pkdb.mayo.edu/variants, accessed on 14 October 2024). Due to the inability to precisely define the deletion edges, this variant cannot be classified according to the American College of Medical Genetics and Genomics (ACMG) guidelines.

**Figure 2:**
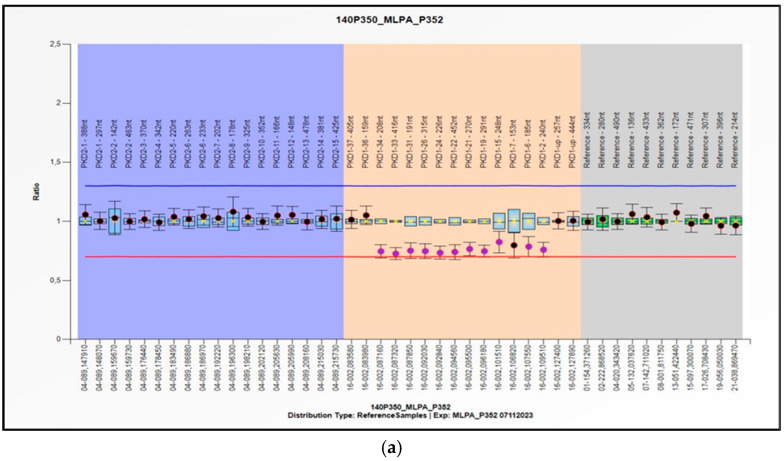

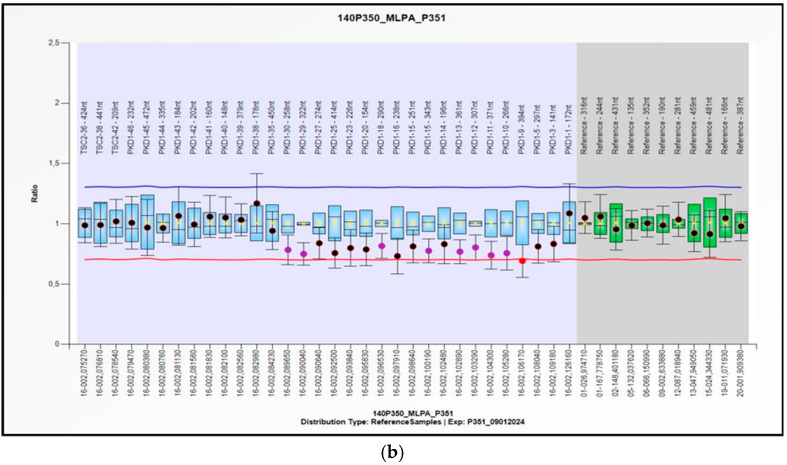
Analysis with multiplex ligation probe amplification (MLPA) of PKD1 gene (light blue and orange background) (NM_001009944.3, except exons 4, 8, 17, 28, 32) and of PKD2 gene (blue background) (NM_000297.4). The molecular investigation was performed on genomic DNA using the revised Kit Salsa MLPA ProbeMix P352-E1 PKD1PKD2 (**a**) and ProbeMix P351-D1 PKD1 ©MRC Holland (**b**). The result shows a deletion of the 2–34 exons of the PKD1 gene in mosaic: NC_000016.10: g. (2136179_2119520) (2097184_2094247) del, chr16:2094247: 2136179: del. (Chr NCBI36/hg18). The arrangement of probes based on chromosomal position reveals a heterozygous deletion (red line): probe ratio <0.5 (red dots), probe ratio 1 < x < 0.5 (purple dots). The first deleted probe was the 240 on the PKD1 gene (hg18 loc.16-002.109510) (**a**), while the last was the 208 on the PKD1 gene (hg18 loc.16-002.087160) (**a**). Black dots represent bounded probes; exons are represented by light blue boxes (green boxes are reference exons). Results are displayed by the Coffalyser.Net software.

A subsequent NGS analysis was performed on a new blood sample, specifically aimed at confirming the presence of mosaicism. Despite this focused approach, the analysis yielded a negative result, aligned with the initial findings. Additionally, a comparative genomic hybridization (CGH) array was conducted to identify potential deletions or duplications in the PKD1 gene, further supporting the MLPA analysis. The CGH array produced a negative outcome, consistent with the technique’s detection threshold for mosaicism at 20%. This test was carried out using the CytoSure^®^ Oligo array ISCA v2 4 × 180 k platform (Oxford Gene Technology, Oxford, UK), with an average resolution of 80 Kb. To reinforce these results, the MLPA analysis was repeated in another laboratory using the same kits, which confirmed our initial findings with identical results. Following a subsequent analysis, the MLPA assay was repeated using an updated version of the kit (SALSA^®^ MLPA^®^ Probemixes P351-D1 PKD1 and P352-E1 PKD1-PKD2). This revision included additional probes that had been absent in the initial analysis, allowing for a more comprehensive coverage of the PKD1 gene. As a result, we were able to accurately delineate the limits of the deleted region, the region from exon 2 to exon 34 (Figure 2): NC_000016.10: g. (2136179_2119520) (2097184_2094247) del; chr16:2094247:2136179: del. (Chr NCBI36/hg18). In support of the presence of mosaicism, the occurrence of at least one single nucleotide variant (SNV) in heterozygosis in the deleted region has been reported.

The variant is not listed in the main ADPKD pathology reference database and cannot be classified according to the ACMG guidelines, since with MLPA it is not possible to establish the exact amino acidic change.

Therefore, given the large deletion and the typical phenotype with nephromegaly and fast-progressing CKD, this variant might be labeled as likely pathogenic.

## 3. Discussion

**Figure 3:**
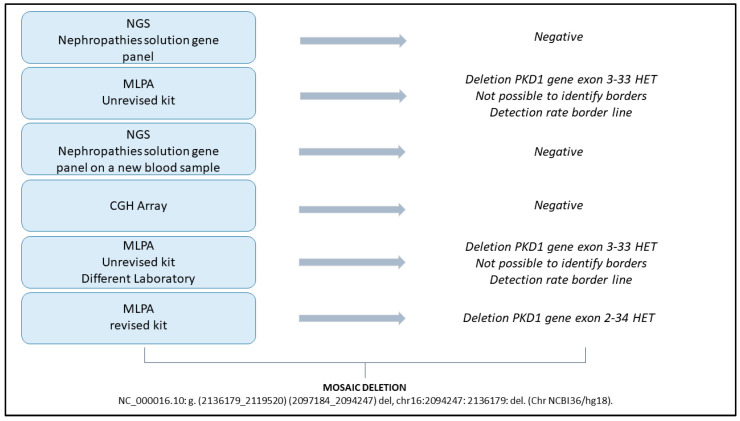
Genetic analysis workflow conducted in sequence.

This clinical case emphasizes the challenges that can arise when attempting genetic analysis in certain cases of ADPKD. The presence of mosaicism, a rare genetic condition where an individual’s cells contain different genomes, has made it particularly challenging to resolve this case. The patient was diagnosed with typical ADPKD based on ultrasound findings, but genetic testing was deemed crucial, not only to assess her eligibility for Tolvaptan therapy but also for the presymptomatic diagnosis of her offspring. Typical cases of ADPKD involve either the PKD1 gene, accounting for 72–75% of solved cases, or the PKD2 gene, accounting for 15–18% of cases. However, 7–10% of the cases are genetically unresolved (GUR) [38]. Atypical cases could be traced back to mutations in other genes, such as GANAB (OMIM #600666) [12], (DNAJB11 OMIM #618061) [13,14], IFT140 (OMIM #614620) [15], ALG5 (OMIM #620056) [16] and ALG 9 (OMIM #606941) [17]. ADPKD is a heterogeneous disease from an allelic point of view. The large deletion of a portion of the PKD1 gene (exons 2–34, Figure 2) in mosaic can be recognized as a complex case of ADPKD. There is a paucity of information on mosaicism and its association with ADPKD [31,32,33,34,35] and a doubtful interpretation of its effect on the pathology. Nevertheless, it is known that mosaicism may occur in at least 1% of ADPKD cases [31]. In this case, several analysis methods were performed that allowed us to determine a definitive diagnosis (Figure 3).

The first method was a targeted NGS gene panel, used for the simultaneous sequencing of numerous genes. The result did not show the presence of any pathogenic variant. The second approach was MLPA, in which most of the exons of PKD1 and PKD2 are simultaneously analyzed for the identification of large rearrangements, duplications, or deletions involving one or more exons or the entire gene not visible when using sequencing techniques. The MLPA analysis showed a large deletion close to the threshold limits, showing that the mutation was in a heterozygous condition. Indeed, as shown in Figure 1 and Figure 2, the probe ratio for the deleted probes remains <1, never reaching the threshold value of 0.5, which would indicate a heterozygous deletion of all cells in the sample. The detection of a signal-to-noise ratio at the resolution limits of the technique suggested that the deletion was in mosaic. Specifically, the deletion encompasses the portion of the PKD1 gene spanning from exon 2 to exon 34. (NC_000016.10: g. (2136179_2119520) (2097184_2094247) del; chr16:2094247: 2136179: del. Chr NCBI36/hg18, revised MLPA kit, Figure 2.) Moreover, the existence of at least one SNV in heterozygosis in the deleted region proves the presence of mosaicism. To confirm and validate the MLPA result, a CGH array was performed, which is a cytogenetic technique used to detect chromosomal abnormalities such as duplications or deletions. The array result was negative, as the mosaic detection threshold was set at 20%.

The variant is not reported in major ADPKD databases. However, a deletion in PKD1 exons 11–15 is reported and classified as pathogenic (https://pkdb.mayo.edu/variants, accessed on 14 October 2024) [20]. Moreover, given the large deletion from exon 2 to exon 34, with PKD1 consisting of 46 exons, and particularly considering the typical phenotype with nephromegaly and rapidly progressive CKD, we may classify the variant as likely pathogenic. In this case, the mutation in mosaic may have contributed to the attenuated reduction in kidney function. Additionally, mosaicism can be suspected in cases of ADPKD without a positive family history. Furthermore, although NGS is considered the most advanced and sensitive technique for detecting mosaicism at the molecular level, in this specific case it was not able to identify the genetic condition. MLPA successfully filled this gap, which NGS was unable to reach.

## 4. Conclusions

In conclusion, targeted sequencing using a gene panel (Targeted NGS testing) remains the preferred first-line diagnostic method for ADPKD, resolving approximately 90–93% of ADPKD cases. This method proves to be particularly effective for patients with a more definite clinical presentation, such as that typical of ADPKD. This approach also facilitates the detection of variants in regions of the PKD1 gene that are prone to high sequence homology. However, it is important to note that 3% of ADPKD cases are associated with large genomic rearrangements, while around 1% are due to mosaicism. In situations where targeted NGS testing does not provide a conclusive diagnosis, second-line strategies, such as MLPA, should be utilized to address these more complex cases.

## Data Availability

The original contributions presented in this study are included in the article. Further inquiries can be directed to the corresponding author.
